# Threat-induced anxiety during goal pursuit disrupts amygdala–prefrontal cortex connectivity in posttraumatic stress disorder

**DOI:** 10.1038/s41398-020-0739-4

**Published:** 2020-02-10

**Authors:** Delin Sun, Andrea L. Gold, Chelsea A. Swanson, Courtney C. Haswell, Vanessa M. Brown, Daniel Stjepanovic, VA Mid-Atlantic MIRECC Workgroup, Jean C. Beckham, Mira Brancu, Patrick S. Calhoun, Eric Dedert, Eric B. Elbogen, Kimberly T. Green, Nathan Kimbrel, Angela Kirby, Gregory McCarthy, Scott D. Moore, Jennifer J. Runnals, Cindy Swinkels, Larry A. Tupler, Elizabeth E. Van Voorhees, Richard D. Weiner, Kevin S. LaBar, Rajendra A. Morey

**Affiliations:** 1 Duke Brain Imaging and Analysis Center, Duke University School of Medicine, Durham, NC USA; 2 Mid-Atlantic Mental Illness Research Education and Clinical Center (MIRECC), Durham, NC USA; 3 Department of Psychiatry and Human Behavior, Warren Alpert Medical School of Brown University, Providence, RI USA; 4 Pediatric Anxiety Research Center, Bradley Hospital, Riverside, RI USA; 5 Department of Psychology, Virginia Polytechnic Institute, Roanoke, VA USA; 6 Department of Psychiatry and Behavioral Sciences, Duke University School of Medicine, Durham, NC USA; 7 Department of Psychology, Yale University, New Haven, CT USA

**Keywords:** Neuroscience, Psychiatric disorders

## Abstract

To investigate how unpredictable threat during goal pursuit impacts fronto-limbic activity and functional connectivity in posttraumatic stress disorder (PTSD), we compared military veterans with PTSD (*n* = 25) vs. trauma-exposed control (*n* = 25). Participants underwent functional magnetic resonance imaging (fMRI) while engaged in a computerized chase-and-capture game task that involved optimizing monetary rewards obtained from capturing virtual prey while simultaneously avoiding capture by virtual predators. The game was played under two alternating contexts—one involving exposure to unpredictable task-irrelevant threat from randomly occurring electrical shocks, and a nonthreat control condition. Activation in and functional connectivity between the amygdala and ventromedial prefrontal cortex (vmPFC) was tested across threat and nonthreat task contexts with generalized psychophysiological interaction (gPPI) analyses. PTSD patients reported higher anxiety than controls across contexts. Better task performance represented by successfully avoiding capture by predators under threat compared with nonthreat contexts was associated with stronger left amygdala–vmPFC functional connectivity in controls and greater vmPFC activation in PTSD patients. PTSD symptom severity was negatively correlated with vmPFC activation in trauma-exposed controls and with right amygdala–vmPFC functional connectivity across all participants in the threat relative to nonthreat contexts. The findings showed that veterans with PTSD have disrupted amygdala–vmPFC functional connectivity and greater localized vmPFC processing under threat modulation of goal-directed behavior, specifically related to successfully avoiding loss of monetary rewards. In contrast, trauma survivors without PTSD relied on stronger threat-modulated left amygdala–vmPFC functional connectivity during goal-directed behavior, which may represent a resilience-related functional adaptation.

## Introduction

Imminent threat elicits fear and is accompanied by phasic fight or flight responses, whereas unpredictable threat is associated with anxiety and sustained hypervigilance and apprehension^1^. Posttraumatic stress disorder (PTSD) is characterized by symptoms of hyperarousal and hypervigilance that produce considerable distress and functional impairment^2^. The most widely adopted behavioral models for studying PTSD are based on fear conditioning and extinction^3,4^, but few studies have examined the brain response associated with the anxiety elicited by the uncertainty resulting from unpredictable threat^5^. Rodents consistently prefer predictable shocks and their associated contexts, and predictability attenuates the negative effects of stress^5^. Rodents exposed to unpredictable threats display a behavioral syndrome akin to PTSD, such as hypervigilance, insomnia, and impaired attention^6,7^. The functional impairment in humans produced by uncertainty is a core feature of anxiety^8^ that is related to PTSD symptoms of avoidance, numbing, and hyperarousal^9,10^. Intolerance to uncertainty is known to predict subsequent PTSD symptoms following campus shootings^9^. Patients with PTSD who are treated with cognitive-behavioral therapy show decreased startle magnitude to unpredictable threat, and this decline is correlated with a decline in PTSD symptoms^11^. Unpredictable threat, frequently experienced by many veterans during deployment, has been linked to impaired goal-directed processing^12,13^, a component of executive functioning, which is compromised upon returning to the demands of civilian life^14^. A central claim of the generalized unsafety theory of stress is that veterans are unable to switch off or inhibit the default stress response, which becomes dependent on the perception of generalized unsafety rather than actual threat^15^.

It is widely accepted that effective regulation of the amygdala response by the prefrontal cortex (PFC), particularly ventromedial PFC (vmPFC)^16^, is crucial for successfully maintaining goal-directed behavior^17^. We previously reported that nonclinical volunteers exposed to unpredictable threat during a computer gaming task elicited greater functional connectivity of right amygdala with vmPFC^12^. Furthermore, right amygdala functional connectivity with vmPFC positively correlated with successful goal-directed behavior during unpredictable threat. Reduced functional connectivity between amygdala and vmPFC has also been documented in PTSD patients regardless of specific task requirements^18,19^.

Our aim was to investigate the functional effects on fronto-limbic systems, particularly the amygdala and vmPFC, when patients with PTSD are exposed to unpredictable threat (unexpected shocks) while simultaneously balancing competing task demands. In this paradigm^12^, participants face threat from unpredictable, task-irrelevant shocks in some task blocks while navigating a virtual avatar through a maze to pursue moving prey and evade pursuit by predators. Prey capture by the avatar and predator capture of the avatar were motivated by monetary gains and losses, respectively. This task design requires active engagement and rapid ongoing response of the participant that imposes identical demands for tracking behavior across unpredictable threat and nonthreat contexts. We hypothesized that better performance on the chase-and-capture game (indexed by either more prey captures or fewer avatar captures) would be associated with greater amygdala–vmPFC functional connectivity in trauma-exposed controls than in PTSD patients. We also hypothesized that modulation of amygdala–vmPFC functional connectivity by threat would be inversely correlated with PTSD symptom severity. In addition, the hippocampus plays a critical role in PTSD^20^ and in encoding contextual information^21^, which is an important component of our task paradigm. We thus hypothesized between-group differences in threat-modulated hippocampal activity and/or connectivity with amygdala/vmPFC. However, we lacked a strong hypothesis about the direction of the comparison given that both decreased^4,22^ and increased^23,24^ hippocampal responses have been reported in patients with PTSD vs. controls.

## Method

### Participants

Twenty-five participants with PTSD and 25 trauma-exposed controls (including three participants with subthreshold PTSD^25^) were recruited from a repository of military service members and veterans who served after September 11, 2001^26^. The sample size was determined by power analyses (Supplementary Data) based on the results of our previous study utilizing the same task paradigm in healthy volunteers^12^, and was larger than the sample size of two recent studies on vmPFC–amygdala functional connectivity in PTSD^18,19^, thus ensures adequate power of detection. Participants underwent screening for inclusion and exclusion criteria based on information available in our repository and from subsequent telephone contact. PTSD diagnosis was confirmed using the Clinician-Administered PTSD Scale for DSM-5^2^ on the day of the scan. Exclusion criteria included major Axis I disorders (other than depressive or anxiety disorders), contraindications to MRI, traumatic brain injury, substance dependence, neurological disorders, and age over 65 years. Participants completed demographic and clinical questionnaires to assess depressive symptoms (Beck Depression Inventory-II, BDI-II)^27^, alcohol use disorder (Alcohol Use Disorders Identification Test)^28^, drug abuse (Drug Abuse Screening Test)^29^, childhood trauma (Child Trauma Questionnaire)^30^, anxiety (State-Trait Anxiety Inventory)^31^, trauma exposure (Traumatic Life Events Questionnaire, TLEQ)^32^, and current medication use. Upon entry into our repository, participants completed the Combat Exposure Scale^33^ as well as the Structured Clinical Interview (SCID) for DSM-IV to assess comorbid Axis I diagnoses. Eleven participants with PTSD and no controls took medications listed in Supplementary Table 1S. All participants provided written informed consent to participate in procedures reviewed and approved by the Institutional Review Boards at Duke University and the Durham Veterans Affairs Medical Center. Participants were compensated $25/h and gained a task bonus of $10–25 based on task performance.

### Experimental procedure

**Fig. 1:**
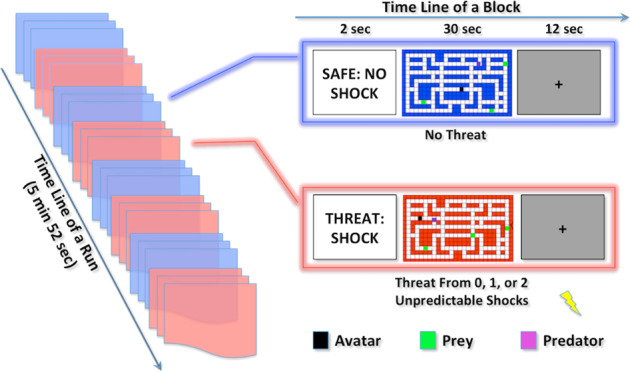
Schematic of the chase-and-capture game paradigm. Participants performed five runs of the task. In each run, there were four threat (represented in red) and four nonthreat (represented in blue) blocks presented in alternating order. In each block, a text cue signaling block type was displayed for 2 s, then followed by a computer game lasting for 30 s, and ended by a fixation cross exhibiting for 12 s. During the computer game, participants were asked to move an avatar (black square) within a 2D maze on the screen through operating joystick to capture prey (green squares) and to avoid capture by a predator (purple square). Prey capture by the avatar and avatar capture by the predator were associated with monetary gain and loss, respectively. No shock was accompanied with the nonthreat block, while there was zero shock in some threat blocks, and one or two shocks in the other threat blocks (at least one shock per run, and on average 0.35 shocks per threat block). The onset of a shock was randomized relative to the onset of the embedded block. The participants had no way to distinguish between threat blocks without shock and threat blocks with unpredictable shocks before the shock delivery.

The chase-and-capture task paradigm (Fig. 1) was described by Gold et al.^12^. Under the task-irrelevant threat of electrical shock, participants used a joystick to navigate an avatar within a 2D maze to capture prey, which generated monetary rewards, and to evade capture by a predator (avatar capture), which incurred monetary loss. The predator, under software control, followed the minimum path with the goal of capturing the avatar.

Prior to the task, the intensity of shock was calibrated for each participant according to his/her tolerance threshold (Supplementary Data). Participants received detailed instructions and completed a practice run. An adaptively-defined difficulty level was utilized to equilibrate task performance of avatar capture across participants. The task difficulty indexed via predator speed from least difficult (level-1) to most difficult (level-5 where predator speed = avatar speed) was modulated by computer program in the practice run. The median value of difficulty was applied and remained constant throughout the MRI task. Task difficulty did not differ between groups (PTSD, 4.3 ± 0.9; controls, 4.5 ± 0.7; *t*(48) = −0.719, *p* = 0.475).

In the MRI task, participants performed five runs of four threat blocks and four nonthreat blocks, which were presented in alternating order per run. No shocks were presented during the nonthreat condition, but unconditional shocks would be delivered at random times during the threat condition. Blocks lasted 30 s and were separated by a 12-s rest period. A cue at the beginning of each block was presented for 2 s to signal an impending threat or nonthreat block. Most threat blocks were allocated one or two shocks, but occasional blocks had no shocks (≥1 shock/run, average = 0.35 shocks/block). The onset of a shock was randomized relative to the onset of the embedded block to minimize collinearity in functional magnetic resonance imaging (fMRI) analyses.

After the experiment, participants self-reported task strategy and anxiety under various contexts: (1) “did you focus more on avoiding predator (=1) or catching prey (=7)?”, (2) “how anxious were you when you entered mazes? 1 = not at all to 9 = highly anxious”, (3) “how anxious were you when staying in mazes? 1 = not at all to 9 = highly anxious”, and (4) “how much did you dread being chased by predator? 1 = not at all to 9 = high dread”.

### Imaging acquisition and preprocessing

Images were acquired on a 3-Tesla GE scanner equipped with an 8-channel headcoil. T1-weighted whole-brain axial images were obtained with 1-mm isotropic voxels using array spatial sensitivity encoding technique and fast spoiled gradient-recall (3D-FSPGR) (TR/TE/flip angle = 8.16 ms/3.18 ms/12°, FOV = 256 mm^2^, 1 mm slice thickness, 172 slices, 256 × 256 matrix). Functional images were obtained using the standard echo-planar pulse sequence (TR/TE/flip angle = 2000 ms/27 ms/60°, FOV = 256 mm^2^, 64 × 64 matrix, 3.8 mm thickness, 34 oblique axial slices, no interslice gap).

Images were preprocessed using the CONN toolbox (https://sites.google.com/view/conn/). After discarding the first three volumes, functional images were slice-time and head-motion corrected, and co-registered to each participant’s structural image. Structural images were segmented, bias corrected and spatially normalized to the Montreal Neurological Institute (MNI) space. The normalization parameters were also applied to normalize the functional images. Finally, functional images were smoothed with an 8-mm FWHM Gaussian kernel.

### Blood-oxygen-level-dependent (BOLD) activation

General Linear Modeling (GLM) with SPM12 (http://www.fil.ion.ucl.ac.uk/spm/) examined BOLD activation. In the individual-level GLM analyses, the regressors for shock occurrences, threat, nonthreat conditions, and nuisance regressors for six head-motion parameters were modeled and convolved with a canonical hemodynamic response function. The shock regressor was modeled with onset in which the shock was delivered and with duration of 0.6 s. The threat and nonthreat condition regressors were modeled with onset of the cue and with the block duration of 32 s (cue = 2 s, plus maze = 30 s). High-pass temporal filtering with a cutoff of 128 s was employed to remove low-frequency drift.

**Fig. 2:**
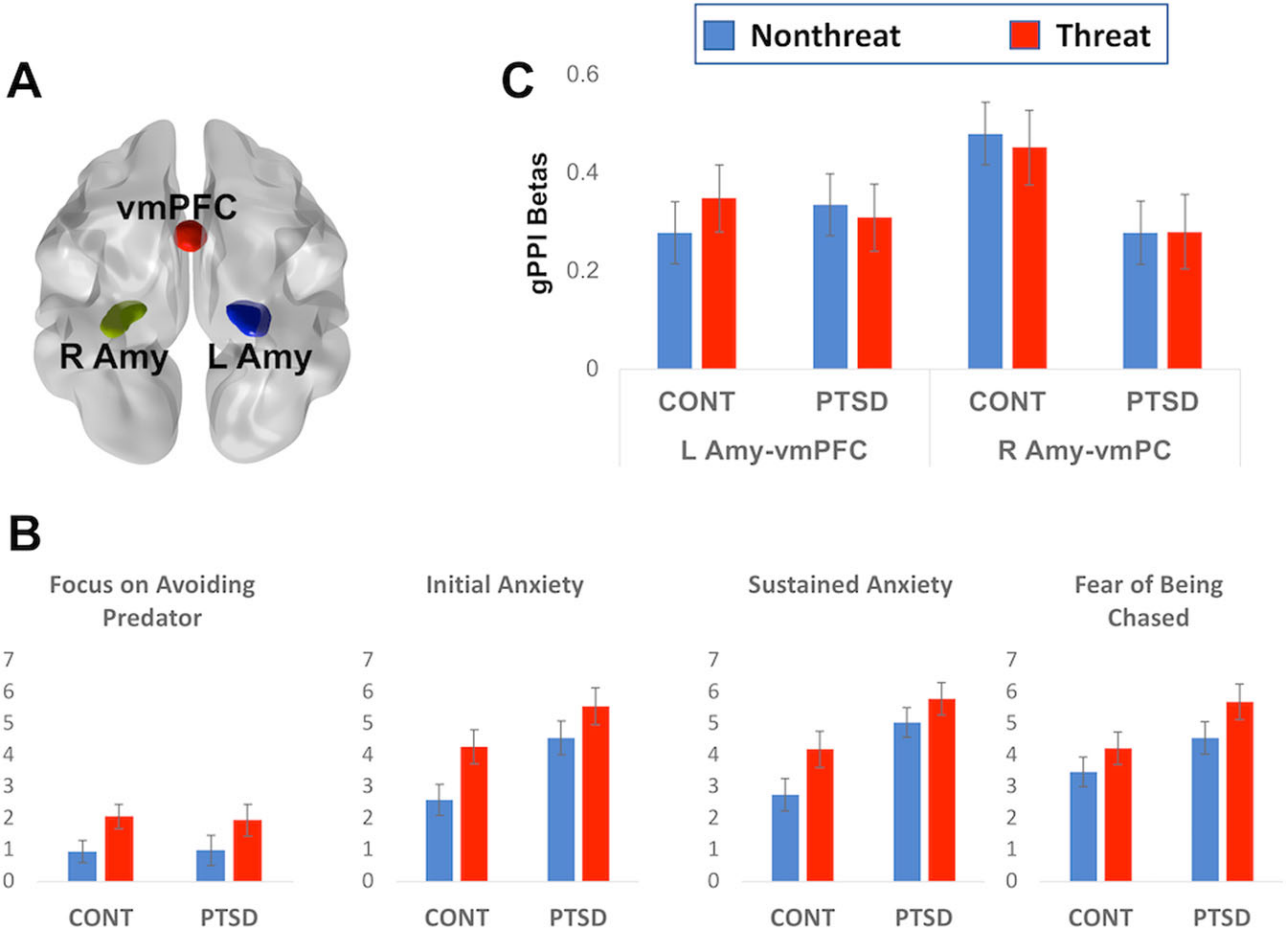
Regions of interest (ROIs), self-report, and functional connectivity results. **a** Three ROIs (bottom view): left amygdala (L Amy), vmPFC and right amygdala (R Amy). **b** Self-report results. Threat vs. nonthreat context elicited enhanced focus on avoiding predator (*F*(1,45) = *7*.181, *p* = 0.010), increased initial (*F*(1,44) = 11.221, *p* = 0.002) and sustained anxiety (*F*(1,44) = 7.469, *p* = 0.009), and enhanced fear of being chased by the predator (*F*(1,44) = 7.299, *p* = 0.010). Participants with PTSD compared with controls (CONT) showed greater initial (*F*(1,44) = 6.266, *p* = 0.016) and sustained (*F*(1,44) = 9.937, *p* = 0.003) anxiety, and a trend of significance of enhanced fear to be chased by the predator (*F*(1,44) = 4.035, *p* = 0.051), but nonsignificant difference in focus on avoiding predator (*F*(1,45) = 0.008, *p* = 0.929). For better understanding, the scores of focus on avoiding predator =7—the scores of the first self-report, since the first self-report question was “did you focus more on avoiding predator (=1) or catching prey (=7)?”. **c** Controls (*F*(1,24) = 5.821, *p* = 0.048) but not participants with PTSD (*F*(1,24) = 0.988, *p* = 0.660) showed stronger right than left amygdala–vmPFC functional connectivity collapsed across contexts. Error bar denotes standard errors of mean.

The group-level statistical inferences were based on mean betas of BOLD responses extracted from the regions of interest (ROI) through MarsBar software (http://marsbar.sourceforge.net). ROIs of bilateral amygdala and hippocampus were defined by the WFU PickAtlas (https://www.nitrc.org/projects/wfu_pickatlas), and the functionally-defined vmPFC ROI (peak MNI coordinates 2, 40, −12) was from our prior study using this paradigm in nonclinical participants^12^ (Fig. 2a).

### Generalized psychophysiological interaction (gPPI)

Functional connectivity was investigated with the generalized gPPI toolbox (https://www.nitrc.org/projects/gppi). We investigated vmPFC correlations with either the left or the right amygdala’s seed time course (physiological regressor) that were significantly modulated by the threat vs. nonthreat task contrast (an interaction of physiological and psychological regressors). We also investigated the functional connections between bilateral hippocampus and bilateral amygdala and vmPFC.

### Statistical analyses

Task performance, self-reports, as well as BOLD activation and functional connectivity extracted from ROIs were each entered into repeated measures analysis of variance (ANOVA) with Group (PTSD vs. controls) as a between-subjects factor and Context (threat vs. nonthreat) as a within-subjects factor. ROIs or seed-target pairs were added as a within-subjects factor if needed. Shapiro–Wilk test, Mauchly’s test, and Levene’s test were employed to test the assumption of normality, sphericity, and equality of variance, respectively (Supplementary Table 2S). The ANOVA model is sensitive to the violation of sphericity but robust to small violation of normality and equality of variance. We thus applied Greenhouse–Geisser method where the assumption of sphericity was violated, and also employed nonparametric methods including Mann–Whitney *U* test for independent samples and Wilcoxon Signed-Ranks test for related samples to validate the statistical significance when the assumption of normality was largely violated (*p* < 0.05 in no less than half of the samples). We did not correct for the violation of equality of variance because it was rare in our data. Results of post hoc analyses were corrected by Bonferroni method. All of the *t*-tests were two-tailed.

#### Task performance and self-reports

Avatar captures and prey captures were recorded every 0.5 s. The average number of prey captures, the average number of avatar captures, and the scores of four self-report questions were each entered into a two-way repeated-measures ANOVA with Group and Context as factors. Four participants with missing data of self-reports were excluded from the corresponding analyses.

#### BOLD activation

The mean betas of brain activity in amygdala and vmPFC were entered into a three-way repeated measures ANOVA with Group, Context, and ROI (left amygdala, right amygdala vs. vmPFC) as factors. The betas from hippocampal activation were entered into a three-way repeated measures ANOVA model with factors for Group, Context, and ROI (left vs. right hippocampus).

The whole-brain voxel-wise analyses of the threat vs. nonthreat contrast and its between-group difference were also conducted. The results were height-thresholded at *p* < 0.001 and subjected to correction of family-wise error (FWE) of *p* < 0.05.

#### Functional connectivity

The mean betas of functional connectivity of vmPFC with bilateral amygdala were entered into a three-way repeated-measures ANOVA with Group, Context, and Seed (left vs. right amygdala) as factors. The mean betas from functional connectivity of bilateral hippocampus with vmPFC and amygdala were entered into a four-way repeated measures ANOVA model with factors for Group, Context, Seed (left amygdala, right amygdala vs. vmPFC), and Target (left vs. right hippocampus).

We also explored the functional connectivity of the left amygdala, right amygdala and vmPFC with the rest of the brain based on voxel-wise analyses in response to the threat vs. nonthreat contrast and its between-group difference. The results were height-thresholded at *p* < 0.001 and FWE correction of *p* < 0.05. For both BOLD and functional analyses, additional ROIs including bilateral inferior frontal gyrus were tested and reported in Supplementary Data.

#### Brain–behavior associations

Pearson’s correlations measured how brain responses (activations or functional connections) to the threat vs. nonthreat contrast were associated with task performance (average number of prey capture or avatar capture) or clinical measures (PTSD severity indexed by current/lifetime CAPS scores or trauma exposure indexed by TLEQ scores). The brain-clinical associations were conducted in both groups to show the full range of symptom severity, rather than restricting the range to patients. The between-group differences of brain–behavior correlations were compared using the Fisher’s *r*-to-*z* transformation^34^ and were corrected by Bonferroni method for the number of ROIs (left amygdala, right amygdala vs. vmPFC) or seed-target pairs (left amygdala–vmPFC vs. right amygdala–vmPFC). The associations between performance or clinical measures and brain activity/connectivity in hippocampus were investigated and corrected for the number of ROIs (left vs. right hippocampus) or seed-target pairs (seed: left amygdala, right amygdala, and vmPFC; target: left vs. right hippocampus). We also reported the correlations between brain responses (activations or functional connections) to either threat or nonthreat context and the corresponding task performance (average number of prey capture or avatar capture).

## Results

### Demographic information

As shown in Table 1, groups did not differ on gender, childhood trauma, substance/alcohol use disorder. PTSD patients compared with controls were slightly older, and had higher levels of trauma exposure, combat exposure, depressive symptoms, and state and trait anxiety. As shown in Supplementary Data, after removing the oldest subject in PTSD patients and the youngest subject in controls, the two groups were not significantly different in age, and showed results consistent with the findings reported in the main text.

**Table 1:** Demographic information and clinical measures.

Test	CONT	PTSD	PTSD vs. CONT	
	Mean (SD)	Mean (SD)	Statistics	*p* value
Sex (M/F)	22/3	22/3	NS	
Age	38.2 (7.8)	43.7 (10.1)	2.143	0.037
CAPS-life	6.3 (6.6)	46.9 (14.7)	12.633	<0.001
CAPS-current	3.1 (1.5)	29.6 (17.9)	7.750	<0.001
BDI-II	4.9 (6.9)	18.2 (13.2)	4.438	<0.001
CTQ	46.0 (24.2)	54.2 (24.5)	1.197	0.237
CES	3.5 (4.7)	14.2 (12.0)	4.144	<0.001
TLEQ	12.7(12.2)	22.1 (10.8)	2.912	0.005
AUDIT	4.0 (3.9)	3.7 (2.8)	−0.345	0.732
DAST	0.2 (0.7)	0.8 (1.2)	1.817	0.077
STAI-state	27.7 (8.4)	40.8 (11.5)	4.578	<0.001
STAI-trait	29.7 (7.9)	44.5 (11.8)	5.197	<0.001

*CONT* trauma-exposed controls, *PTSD* PTSD patients, *CAPS-life* Clinician-Administered PTSD Scale reflecting symptoms in the worst 30-day period of subject’s life, *CAPS-current* Clinician-Administered PTSD Scale reflecting symptoms in the past month, *BDI* Beck Depression Inventory-II, *CTQ* Child Trauma Exposure questionnaire, *CES* Combat Exposure Scale, *TLEQ* Traumatic Life Events Questionnaire, *AUDIT* Alcohol Use Disorders Identification Test, *DAST* Drug Abuse Screening Test, *STAI-state* state anxiety of State-Trait Anxiety Inventory, *STAI-trait* trait anxiety of State-Trait Anxiety Inventory, *NS* no significance.

### Task performance

The average number of prey capture did not differ by Context (*F(*1,48) = 0.489, *p* = 0.488) or the Group × Context interaction (*F*(1,48) = 0.088, *p* = 0.768), but showed a trend towards significance for Group (*F*(1,48) = 3.164, *p* = 0.082) depicting that controls (mean ± SD: nonthreat, 6.229 ± 0.977; threat, 6.271 ± 1.136) captured more preys than participants with PTSD (mean ± SD: nonthreat, 5.543 ± 1.507; threat, 5.646 ± 1.674).

The average number of avatar capture did not differ by Group (*F*(1,48) = 0.422, *p* = 0.519) and Context (*F*(1,48) = 1.103, *p* = 0.299), but revealed a Group × Context interaction (*F*(1,48) = 5.823, *p* = 0.020). Post hoc analyses found no between-context difference in participants with PTSD (mean ± SD: nonthreat, 1.403 ± 0.785; threat, 1.303 ± 0.707; *t*(24) = −1.017, *p* = 0.590), but showed a trend towards significance in which controls were less captured in nonthreat than threat context (mean ± SD: nonthreat, 1.093 ± 0.638; threat, 1.350 ± 0.893; *t*(24) = 2.246, *p* = 0.068; Wilcoxon Signed-Ranks test, significance = 0.072).

### Self-report assessments

As shown in Fig. 2b, the main effect of Context showed that threat relative to nonthreat context elicited enhanced focus on avoiding predator relative to capturing prey (*F*(1,45) = *7*.181, *p* = 0.010 Wilcoxon Signed-Ranks test, significance = 0.014), increased initial (*F*(1,44) = 11.221, *p* = 0.002 Wilcoxon Signed-Ranks test, significance = 0.001) and sustained anxiety (*F*(1,44) = 7.469, *p* = 0.009 Wilcoxon Signed-Ranks test, significance = 0.005), and enhanced fear of being chased by the predator (*F*(1,44) = 7.299, *p* = 0.010 Wilcoxon Signed-Ranks test, significance = 0.011). The main effect of Group showed that participants with PTSD compared with controls showed greater initial (*F*(1,44) = 6.266, *p* = 0.016; Mann–Whitney *U* test, significance = 0.021) and sustained (*F*(1,44) = 9.937, *p* = 0.003; Mann–Whitney *U* test, significance = 0.006) anxiety, and a trend of enhanced fear to be chased by the predator (*F*(1,44) = 4.035, *p* = 0.051; Mann–Whitney *U* test, significance = 0.058), but non difference in focus on avoiding predator (*F*(1,45) = 0.008, *p* = 0.929; Mann–Whitney *U* test, significance = 0.716). No Group × Context interactions were observed (*F* values < 0.8, *p* values > 0.4; Mann–Whitney *U* test, all significance > 0.4).

### BOLD activation

For BOLD activation in amygdala and vmPFC, the significant main effect of Context showed that threat vs. nonthreat context elicited larger activation across ROIs in both groups (*F*(1,48) = 5.330, *p* = 0.025). The significant main effect of ROI (*F*(2,88) = 9.259, *p* = 0.002) indicated larger activation in both left (*F*(1,48) = 10.944, *p* = 0.006) and right (*F*(1,48) = 9.113, *p* = 0.012) amygdala than vmPFC, whereas no significant difference between left and right amygdala (*F*(1,48) = 0.24, *p* > 1). The main effect of Group (*F*(1,48) = 0.002, *p* = 0.961), Group × Context interaction (*F*(1,48) = 2.141, *p* = 0.150), Group × ROI (*F*(2,96) = 0.191, *p* = 0.719), Group × Contex × ROI (*F*(2,96) = 0.134, *p* = 0.827), and Context × ROI (*F*(2,96) = 1.638, *p* = 0.205) were nonsignificant.

For the hippocampal activation, there was a significant Group × Context interaction (*F*(1,48) = 5.550, *p* = 0.023), depicting that the threat vs. nonthreat contrast elicited smaller activation in bilateral hippocampus in participants with PTSD compared with controls, although further analyses revealed no between-group difference under either threat or nonthreat context (*F* values *<* 1.7*, p* values *>* 0.4). No other effects were significant (*F* values < 1.2, *p* values > 0.2).

Whole-brain voxel-wise analyses showed that threat compared with nonthreat context was associated with lower activation in the bilateral lingual gyrus (peak *Z* value = 3.91, cluster size = 715 voxels, peak coordinates *x* = 0, *y* = −72, *z* = 12). Neither main effect of Group nor the Group × Context interaction was detected significant.

### Functional connectivity

For amygdala–vmPFC functional connectivity, independent of task context, the significant Seed × Group interaction (*F*(1,48) = 6.515, *p* = 0.014) indicated larger right than left amygdala–vmPFC functional connectivity in controls (*F*(1,24) = 5.821, *p* = 0.048, Fig. 2c) but not in participants with PTSD (*F*(1,24) = 0.988, *p* = 0.660). The main effect of Group (*F*(1,48) = 1.200, *p* = 0.279), Group × Context interaction (*F*(1,48) = 0.318, *p* = 0.576), Seed (*F*(1,48) = 2.044, *p* = 0.159), Context (*F*(1,48) = 0.020, *p* = 0.889), and Seed × Context interaction (*F*(1,48) = 1.054, *p* = 0.310) were all nonsignificant.

Hippocampal functional connectivity with amygdala or vmPFC showed a significant main effect of Seed (*F*(2,96) = 37.028, *p* < 0.001), where hippocampal functional connectivity with both left (*F*(1,48) = 74.430, *p* < 0.001) and right amygdala (*F*(1,48) = 30.001, *p* < 0.001) were larger than hippocampus–vmPFC functional connectivity across contexts and groups. There was a trend for greater left compared to right amygdala functional connectivity (*F*(1,48) = 5.393, *p* = 0.075) with hippocampus. None of the other effects was significant for hippocampal functional connectivity (*F* values < 2.8, *p* values > 0.1).

Whole-brain voxel-wise analyses investigating the functional connectivity of the left amygdala, right amygdala, and vmPFC with the rest of the brain detected neither main effect of Group nor a Group × Context interaction.

### Brain-performance associations

**Fig. 3:**
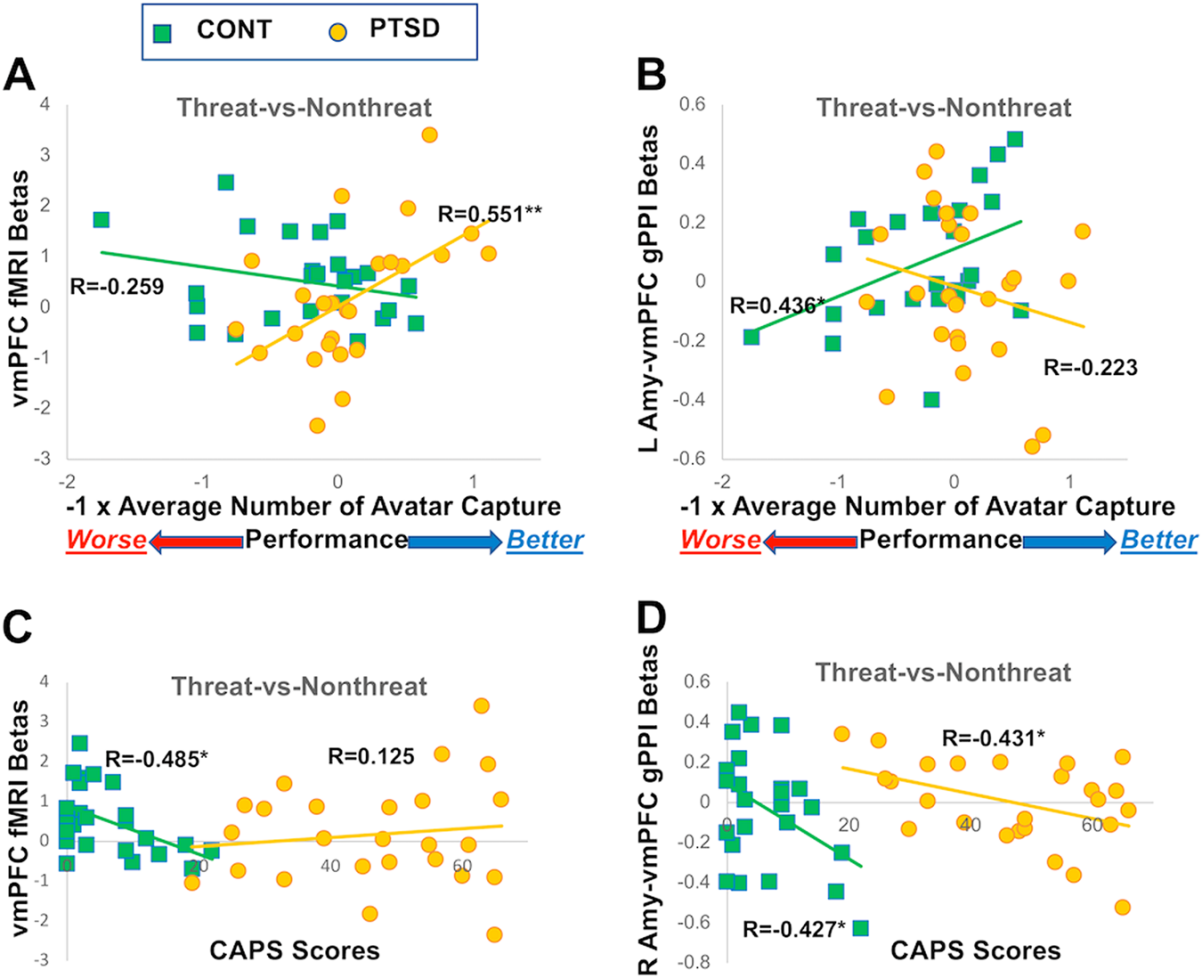
Brain-performance and brain-clinical associations. For the threat vs. nonthreat contrast, better performance (i.e., fewer avatar captures) is associated with (**a**) larger vmPFC activation in PTSD (*R* *=* 0.551, *p* = 0.004) but not in controls (*R* *=* −0.259, *p* = 0.211; two correlations were significantly different, Fisher’s *z* *=* 2.935, *p* = 0.009), and (**b**) stronger left amygdala–vmPFC functional connectivity in trauma-exposed controls (*R* *=* 0.436, *p* = 0.029) but not in participants with PTSD patients (*R* *=* −0.223, *p* = 0.285; two correlations were significantly different, Fisher’s *z* *=* 2.301, *p* = 0.042). For observation purpose only, the *x*-axis reflects −1 × average number of avatar capture, so that higher values represent better performance. CAPS scores were negatively correlated with (**c**) vmPFC activity in controls (*R* = −0.485, *p* = 0.014) but not PTSD participant (*R* = 0.125, *p* = 0.553; two correlations were different at a trend level, Fisher’s *z* = −2.173, *p* = 0.090), and (**d**) right amygdala–vmPFC functional connectivity in both PTSD (*R* = -0.431, *p* = 0.032) and CONT (*R* = −0.427, *p* = 0.033) groups, in response to the threat vs. nonthreat contrast. *p < 0.05; **p < 0.005.

For the threat vs. nonthreat contrast, as shown in Fig. 3a, better performance indexed by fewer avatar captures was accompanied with larger vmPFC activation in PTSD patients (*R* = 0.551, *p* = 0.004) but not in controls (*R* = -0.259, *p* = 0.211), and the two correlations were significantly different (Fisher’s *z* *=* 2.935, *p* = 0.009). As shown in Fig. 3b, better performance (i.e., fewer avatar captures) also related to stronger left amygdala–vmPFC functional connectivity in controls (*R* = 0.436, *p* = 0.029) but not in PTSD patients (*R* = -0.223, *p* = 0.285), and the two correlations were significantly different (Fisher’s *z* *=* 2.301, *p* = 0.042). The number of prey capture did not correlate with either brain activation or functional connections (*p* values > 0.1). Correlations between performance and brain response in either threat or nonthreat context are reported in Table 2.

**Table 2:** Task performance correlates with brain activation per ROI and functional connectivity (gPPI) per seed.

Performance	Condition	PTSD [R(p)]	CONT [R(p)]	PTSD vs. CONT *p* value
vmPFC				
Prey capture	Threat	0236 (0.255)	−0.082 (0.695)	0.283
	Nonthreat	0.175 (0.404)	−0.304 (0.139)	0.103
Avatar capture	Threat	−0.261 (0.207)	−0.300 (0.145)	0.889
	Nonthreat	−0.198 (0.344)	−0.368 (0.070)	0.537
L Amygdala				
Prey capture	Threat	0.116 (0.582)	−0.317 (0.122)	0.140
	Nonthreat	0.143 (0.495)	−0.514 (0.009)	0.018
Avatar capture	Threat	0.157 (0.453)	−0.411 (0.041)	0.048
	Nonthreat	0.174 (0.406)	−0.505 (0.010)	0.015
R Amygdala				
Prey capture	Threat	−0.094 (0.655)	−0.308 (0.134)	0.457
	Nonthreat	−0.090 (0.668)	−0.398 (0.049)	0.273
Avatar capture	Threat	−0.074 (0.726)	−0.067 (0.749)	0.983
	Nonthreat	0.149 (0.477)	−0.188 (0.368)	0.259
Left amygdala–vmPFC functional connectivity				
Prey capture	Threat	0.158 (0.450)	−0.251 (0.226)	0.168
	Nonthreat	−0.001 (0.996)	−0.053 (0.802)	0.864
Avatar capture	Threat	−0.011 (0.959)	−0.124 (0.555)	0.706
	Nonthreat	0.130 (0.535)	−0.077 (0.714)	0.490
Right amygdala–vmPFC functional connectivity				
Prey capture	Threat	0.209 (0.317)	−0.263 (0.203)	0.110
	Nonthreat	−0.087 (0.680)	−0.055 (0.795)	0.915
Avatar capture	Threat	0.172 (0.412)	−0.140 (0.503)	0.297
	Nonthreat	0.226 (0.278)	0.069 (0.744)	0.626

*R* and *p* values were based on Pearson correlation within either trauma-exposed controls (CONT) or PTSD patients (PTSD). The between-group comparisons were based on fisher’s *r*-to-z transformation, and the *p* values were uncorrected.

### Brain-clinical associations

For the threat vs. nonthreat contrast, higher lifetime CAPS score was accompanied with smaller vmPFC activation in controls (*R* = −0.485, *p* = 0.014, Fig. 3c) but not in PTSD patients (*R* = 0.125, *p* = 0.553), and the two correlations showed a trend-level difference (Fisher’s *z* = −2.173, *p* = 0.090). Higher lifetime CAPS score was also associated with smaller right amygdala–vmPFC functional connectivity in both controls (*R* = −0.427, *p* = 0.033) and PTSD patients (*R* = −0.431, *p* = 0.032), Fig. 3d, whereas the two correlations were not significantly different (Fisher’s *z* = 0.016, *p* = 0.987). Lifetime CAPS score was related with neither brain activation in amygdala (*p* values > 0.1) nor the left amygdala–vmPFC functional connectivity in both groups (*p* values > 0.1). No significant associations were found between current CAPS score and brain activity or brain connectivity (*p* values > 0.1).

No significant correlation was found in either group between TLEQ score and threat-modulated activation in amygdala and vmPFC or functional connectivity between amygdala and vmPFC (*p* values > 0.2). For the aforementioned statistical outputs, supplemental statistical models involving covariates for gender, childhood trauma, alcohol abuse, and drug abuse yielded consistent findings (Supplementary Data). TLEQ score was positively associated with threat-modulated functional connectivity between right amygdala and right hippocampus in PTSD (*R* = 0.444, *p* = 0.026) but not control (*R* = −0.342, *p* = 0.095) subjects. The two correlations were significantly different (Fisher’s *z* *=* 2.764, *p* = 0.036) indicating an interaction of trauma exposure with PTSD diagnosis.

## Discussion

Here we investigated how task-irrelevant, unpredictable threats impacted fronto-limbic circuitry during goal pursuit in recent war veterans with PTSD and trauma-exposed controls. We used an engaging chase-and-capture game that involved both obtaining monetary rewards and avoiding monetary losses based on task performance. Thus, the task required both vigilance to avoid predators (monetary losses) and pursuit of prey (rewards), which placed demands on attentive, motivational, and cognitive capacities, particularly in the context of task-irrelevant threats. Participants received unpredictable shocks during the threat blocks. The intensity of the shocks was custom calibrated for each participant before starting the formal task to a voltage level the participant deemed to be “highly annoying, but not painful.” This procedure has been used successfully in previous studies without untoward consequences^35,36^. PTSD patients relative to trauma-exposed veterans reported feeling more anxious overall in the task, but both groups reported that the threat context enhanced focus on avoiding predator, increased initial and sustained anxiety, and enhanced fear of being chased by the predator. Consistent with our a priori hypotheses, the results showed that the better performance represented by the successful avoidance of avatar capture by predators was associated with stronger threat-modulated functional connectivity between left amygdala and vmPFC in controls but not in PTSD. In addition, the successful avoidance of avatar capture by predators was associated with heightened threat-modulated regional activation in the vmPFC in PTSD but not in controls. In addition, we found a negative correlation between PTSD severity and threat-modulated functional connectivity between the right amygdala and vmPFC in both groups, and a negative correlation between PTSD severity and threat-modulated vmPFC activation in controls only.

Threat-modulated association between performance and amygdala–vmPFC functional connectivity differed as a function of PTSD (Fig. 3b). This is consistent with the dominant theory that PTSD is accompanied with aberrant amygdala–vmPFC circuitry^16^. This study is the first to investigate functional connectivity associated with unpredictable threat-induced anxiety in PTSD during goal-directed actions. While the functional connectivity findings in PTSD may reflect impaired top-down inhibition of the amygdala by vmPFC^37^, it may also reflect disrupted bottom-up modulation of vmPFC by the amygdala, or dysregulated bidirectional modulation. In both cases, cortical–subcortical interactions, which have previously been implicated in regulating negative emotions^38^ and maintaining neural functions required for goal-directed behavior^12^, are challenged by unpredictable environmental threats in PTSD. However, gPPI is a correlational method, and advanced, directional modeling methods are needed to uncover the direction of functional connectivity between the amygdala and vmPFC in PTSD. In contrast to the veterans with PTSD, trauma-exposed controls show stronger left amygdala–vmPFC functional connectivity related to predator capture to accomplish the task, which may represent a posttrauma functional adaptation and/or a pretrauma resilience marker.

The present left-lateralization of threat-modulated task performance association with amygdala–vmPFC functional connectivity in controls is consistent with reports that harm avoidance is associated with left amygdala–vmPFC functional connectivity in healthy subjects^39^. However, risk tolerance^40^ and reward processing^12^ are associated with right amygdala–vmPFC functional connectivity, suggesting a functional dissociation between the two connections. These differences suggest that the amygdala–vmPFC functional connections are lateralized by contextual information, and the downstream implications of threat such as risk, loss, and harm, but also by the potential for profit. The present finding shows that PTSD further modulates this calculus to influence lateralization of amygdala–vmPFC functional connectivity.

Furthermore, PTSD severity was inversely related to threat-modulated functional connectivity between right amygdala and vmPFC in both groups of participants. Optogenetic activation of amygdala afferents to the rodent infralimbic cortex increases anxiety^41^, while inhibiting these afferents has anxiolytic effects. It is possible that veterans with more severe PTSD symptoms in the present study may have inhibited the bottom-up afferents from right amygdala to vmPFC to a larger extent to diminish the interference of anxiety on goal-directed actions. Our finding that brain connectivity during explicit task engagement can predict PTSD symptoms may have potential clinical applications to help in the diagnosis and treatment monitoring of PTSD. This result remains significant when controlling for clinical confounds either by adding the relevant covariates or excluding the affected subjects (Supplementary Data).

In contrast to most previously published reports that used fear conditioning paradigms to study phasic anxiety and its neural response, the present study engaged patients with PTSD in a dynamic goal-directed task coupled with exposure to unpredictable threat^42^. In trauma-exposed veterans resilient to PTSD, exposure to unpredictable threat produces sustained subjective anxiety^12^, whereas predictable threat elicits a phasic fear response with a lower level of sustained anxiety^43^. As compared with generalized anxiety disorder, patients with PTSD experience greater sustained anxiety from exposure to unpredictable threat^44^. In fact, the startle reaction to unpredictable threat is a better proxy than predictable threat for treatment success of PTSD and other fear-based disorders such as panic and social anxiety disorder^11^. The persistent inability to perceive safety produces a generalized response following prolonged exposure to unpredictable threat and PTSD^45^. Veterans show greater sensitivity to prediction errors for negative outcomes, and higher PTSD symptom severity is accompanied with lower value-tracking in the amygdala^46^.

Prior research links PTSD to hypoactivation in vmPFC and hyperactivation in amygdala during negative emotion processing^16^. Here we find significant performance-related between-group differences in vmPFC activity but not in amygdala activity. Our participants appear to employ different strategies that impact the utilization of vmPFC, and vmPFC functional connections to amygdala, to effect performance differences. The vmPFC plays a crucial role in fear extinction and extinction retention^16^, and PTSD is associated with decreased vmPFC activation in response to threat or stress^18^. US Special Forces who are resilient to severe trauma display stronger vmPFC activation in response to the expectation of reward^47^. Furthermore, PTSD patients with robust vmPFC activation are better able to associate previously encountered aversive stimuli with safety than patients with weak vmPFC activation^48^. In line with these previous findings, stronger vmPFC activation might contribute to fulfilling goal-directed tasks by inhibiting conditioned fear responses to unpredictable threats. It is possible that veterans with PTSD place a greater reliance on local processing in the vmPFC to compensate for amygdala–vmPFC functional disconnectivity.

We also investigated threat-modulated brain activation in hippocampus as well as amygdala–hippocampus and vmPFC–hippocampus functional connectivity, given the critical role of hippocampus in PTSD^20^ and in processing contextual information^21^, which is an important component of our task paradigm. We found that the threat-modulated brain activation in bilateral hippocampi was weaker in patients with PTSD than controls. Previous studies showed that hippocampal activation positively correlates with the probability of threat^49,50^. Our result implies that the hippocampal response of PTSD patients does not selectively discriminate between threat and nonthreat contexts. Moreover, we found a positive relationship between TLEQ score and threat-modulated functional connectivity between right amygdala and right hippocampus that was significantly greater in PTSD patients than in controls. Relatedly, an impaired ability to discriminate between emotional experiences is accompanied by enhanced alpha synchrony between the amygdala and hippocampus^51^. Our findings may reflect that the failure to discriminate between threat and nonthreat contexts is amplified by the magnitude of trauma exposure in PTSD. However, we did not find any relationship between task performance and hippocampal activation or hippocampal functional connection with amygdala/vmPFC. Thus, as compared with vmPFC and amygdala, the hippocampus plays a limited role in task performance, which requires balancing competing task demands during simultaneous exposure to unpredictable threat.

### Limitations and strengths

There are two strengths of this study. First, although the PTSD group showed higher levels of trauma exposure, trauma exposure was not related to regional activation or connectivity measures in amygdala and vmPFC, suggesting the results in these areas cannot be simply explained by magnitude of trauma exposure. Second, the most important findings of the brain-performance and brain-clinical associations are not based on the threat or nonthreat context separately but the threat vs. nonthreat contrast, which is better to reflect the inner processes of anxiety induced by the unpredictable threat without the confounding effects such as motion. There are also three shortcomings in the present study. First, there is no predictable threat condition to serve as a control condition. Second, the PTSD patients are slightly older than the trauma-exposed controls, but reanalyses on a subset of participants matched for age, showed comparable results (Supplementary Data). Third, a nontraumatized control group is lacking to further explore the modulation of brain activity and connectivity by trauma exposure. We previously applied the same task paradigm in nonclinical volunteers^12^ and found greater threat-modulated right amygdala–vmPFC functional connectivity, as well as a positive correlation between average prey captures (i.e., monetary gains) and right amygdala–vmPFC functional connectivity under the threat context. Although the veterans recruited in the present study were different from the healthy participants in the previous study^12^ in not only trauma exposure but also several demographic measures including age, the different patterns of functional connectivity raise the possibility that trauma exposure influences strategies for goal-directed behaviors. Thus, future work could test our hypothesis that people without trauma exposure are more driven by reward anticipation, whereas those (with or without PTSD) with trauma exposure are more driven by loss aversion.

## Conclusion

Veterans with PTSD showed disrupted amygdala–vmPFC functional connectivity and place greater reliance on localized vmPFC processing under threat modulation of goal-directed behavior, specifically related to successful task performance while avoiding loss of monetary rewards. On the other hand, trauma survivors without PTSD rely on stronger left amygdala–vmPFC functional connectivity during goal-directed behavior that is modulated by threat, which may represent a resilience-related functional adaptation.

## Supplementary information

Supplementary Data

Supplementary Table 1S

Supplementary Table 2S

## Data Availability

All of the raw data are available upon request to the corresponding author.

## Code availability

All of the computer codes are available upon request to the corresponding author.
