# Supplementary material for: Threat-induced anxiety during goal pursuit disrupts amygdala–prefrontal cortex connectivity in posttraumatic stress disorder
Source: Transl Psychiatry. 2020 Feb 10;10:61. doi: 10.1038/s41398-020-0739-4 (PMC7026403; doi:10.1038/s41398-020-0739-4)
Supplement: Supplementary file 2 — Supplementary Table 1S [file 41398_2020_739_MOESM2_ESM.docx]

# **Table 1S**. Psychotropic medications taken by PTSD patients daily.

| Patient ID | Medication 1 | Medication 2 | Medication 3 |
| --- | --- | --- | --- |
| 1 | Trazodone | Klonopin | Zoloft |
| 2 | Methylphenidate | Wellbutrin | - |
| 3 | Citalopram | Prozac | - |
| 4 | Clonazepam | Citalopram | - |
| 5 | Trazodone | - | - |
| 6 | Sertraline | - | - |
| 7 | Cymbalta | - | - |
| 8 | Venlafaxine | - | - |
| 9 | Mirtazapine | - | - |
| 10 | Sertraline | - | - |
| 11 | Citalopram | - | - |
| 12-25 | - | - | - |
