# Supplementary material for: Threat-induced anxiety during goal pursuit disrupts amygdala–prefrontal cortex connectivity in posttraumatic stress disorder
Source: Transl Psychiatry. 2020 Feb 10;10:61. doi: 10.1038/s41398-020-0739-4 (PMC7026403; doi:10.1038/s41398-020-0739-4)
Supplement: Supplementary file 3 — Supplementary Table 2S [file 41398_2020_739_MOESM3_ESM.docx]

# **Table 2s.** Statistical significance for tests of normality, sphericity and equality of variances.

|  | **Shapiro-Wilk** | | **Mauchly's** | **Levene's** |
| --- | --- | --- | --- | --- |
|  | **CONT** | **PTSD** |  |  |
| ***Task Performance*** | | | | |
| Threat, Number of Prey Capture | 0.849 | 0.493 | na | 0.109 |
| NonThreat, Number of Prey Capture | 0.810 | 0.341 |  | 0.062 |
|  |  |  |  |  |
| ***Task Performance*** | | | |  |
| Threat, Number of Avatar Capture | <0.001 | 0.273 | na | 0.874 |
| NonThreat, Number of Avatar Capture | 0.033 | 0.104 |  | 0.481 |
|  |  |  |  |  |
| ***Self Report*** | | | | |
| Threat, focus on predator or prey | <0.001 | <0.001 | na | 0.940 |
| NonThreat, focus on predator or prey | <0.001 | <0.001 |  | 0.710 |
|  |  |  |  |  |
| ***Self Report*** | | | | |
| Threat, anxious when entering maze | 0.050 | 0.015 | na | 0.366 |
| NonThreat, anxious when entering maze | <0.001 | 0.039 |  | 0.049 |
|  |  |  |  |  |
| ***Self Report*** | | | | |
| Threat, anxious when in maze | 0.029 | 0.011 | na | 0.421 |
| NonThreat, anxious when in maze | <0.001 | 0.009 |  | 0.010 |
|  |  |  |  |  |
| ***Self Report*** | | | | |
| Threat, dread being chased | 0.088 | 0.023 | na | 0.274 |
| NonThreat, dread being chased | 0.015 | 0.183 |  | 0.651 |
|  |  |  |  |  |
|  | ***fMRI*** |  |  |  |
| Threat, L Amy | 0.805 | 0.975 | Context, na;  ROI, < 0.001;  Context x ROI, 0.001 | 0.367 |
| Threat, R Amy | 0.644 | 0.100 |  | 0.442 |
| Threat, vmPFC | 0.611 | 0.237 |  | 0.094 |
| NonThreat, L Amy | 0.600 | 0.358 |  | 0.630 |
| NonThreat, R Amy | 0.913 | 0.669 |  | 0.514 |
| NonThreat, vmPFC | 0.341 | 0.117 |  | 0.024 |
|  |  |  |  |  |
|  | ***fMRI*** |  |  |  |
| Threat, L Hip | 0.938 | 0.657 | na | 0.892 |
| Threat, R Hip | 0.226 | 0.753 |  | 0.348 |
| NonThreat, L Hip | 0.731 | 0.670 |  | 0.487 |
| NonThreat, R Hip | 0.110 | 0.960 |  | 0.203 |
|  |  |  |  |  |
|  | ***gPPI*** |  |  |  |
| Threat, L Amy to vmPFC | 0.253 | 0.942 | na | 0.649 |
| Threat, R Amy to vmPFC | 0.679 | 0.969 |  | 0.121 |
| NonThreat, L Amy to vmPFC | 0.624 | 0.611 |  | 0.313 |
| NonThreat, R Amy to vmPFC | 0.453 | 0.905 |  | 0.014 |
|  |  |  |  |  |
|  | ***gPPI*** |  |  |  |
| Threat, L Amy to L Hip | 0.571 | 0.567 | Context, na;  Seed, na;  Target, 0.135;  Context x Target, na;  Context x Seed, 0.002;  Seed x Target, 0.208;  Context x Seed x Target, 0.579. | 0.902 |
| Threat, R Amy to L Hip | 0.634 | 0.334 |  | 0.324 |
| Threat, vmPFC to L Hip | 0.233 | 0.061 |  | 0.484 |
| Threat, L Amy to R Hip | 0.658 | 0.384 |  | 0.331 |
| Threat, R Amy to R Hip | 0.728 | 0.801 |  | 0.925 |
| Threat, vmPFC to L Hip | 0.599 | 0.137 |  | 0.724 |
| NonThreat, L Amy to L Hip | 0.794 | 0.390 |  | 0.871 |
| NonThreat, R Amy to L Hip | 0.467 | 0.573 |  | 0.090 |
| NonThreat, vmPFC to L Hip | 0.740 | 0.434 |  | 0.710 |
| NonThreat, L Amy to R Hip | 0.192 | 0.037 |  | 0.745 |
| NonThreat, R Amy to R Hip | 0.632 | 0.430 |  | 0.925 |
| NonThreat, vmPFC to L Hip | 0.940 | 0.672 |  | 0.355 |

Note : Amy = amygdala, Hip = hippocampus, vmPFC = ventromedial prefrontal cortex, L = left, R = right.
